# Prehospital volume resuscitation - Did evidence defeat the crystalloid dogma? An analysis of the TraumaRegister DGU® 2002–2012

**DOI:** 10.1186/s13049-016-0233-4

**Published:** 2016-04-06

**Authors:** Arne Driessen, Matthias Fröhlich, Nadine Schäfer, Manuel Mutschler, Jerome M. Defosse, Thomas Brockamp, Bertil Bouillon, Ewa K. Stürmer, Rolf Lefering, Marc Maegele, the TraumaRegister DGU

**Affiliations:** Department of Orthopaedic Surgery, Traumatology and Sports Traumatology, Cologne-Merheim Medical Centre (CMMC), Witten/Herdecke University, Ostmerheimer Str. 200, D-51109 Cologne, Germany; Department of Medicine, Institute for Research in Operative Medicine, Faculty of Health, Witten/Herdecke University, Ostmerheimer Str. 200, D-51109 Cologne, Germany; Department of Anaesthesiology and Intensive Care Medicine, Cologne-Merheim Medical Centre, Witten/Herdecke University, Ostmerheimer Str. 200, D-51109 Cologne, Germany

**Keywords:** Pre-hospital volume administration, Coagulopathy, Massive transfusion, Crystalloids, Colloids, Hyperoncotic fluids, Trauma care

## Abstract

**Background:**

Various studies have shown the deleterious effect of high volume resuscitation following severe trauma promoting coagulopathy by haemodilution, acidosis and hypothermia. As the optimal resuscitation strategy during prehospital trauma care is still discussed, we raised the question if the amount and kind of fluids administered changed over the recent years. Further, if less volume was administered, fewer patients should have arrived in coagulopathic depletion in the Emergency Department resulting in less blood product transfusions.

**Methods:**

A data analysis of the 100 489 patients entered into the TraumaRegister DGU® (TR-DGU) between 2002 and 2012 was performed of which a total of 23512 patients (23.3 %) matched the inclusion criteria. Volume and type of fluids administered as well as outcome parameter were analysed.

**Results:**

Between 2002 and 2012, the amount of volume administered during prehospital trauma care decreased from 1790 ml in 2002 to 1039 ml in 2012. At the same time higher haemoglobin mean values, higher Quick’s mean values and reduced mean aPTT can be observed. Simultaneously, more patients received catecholamines (2002: 9.2 to 2012: 13.0 %). Interestingly, the amount of volume administered decreased steadily regardless of the presence of shock. Fewer patients were in the need of blood products and the number of massive transfusions (≥10 pRBC) more than halved.

**Discussion:**

The changes in volume therapy might have reduced haemodilution potentially resulting in an increase of the Hb value. During the period observed transfusion strategies have become more restrictiveand ratio based; the percentage of patients receiving MT halved as blood products may imply negative secondary effects. Furthermore, preventing administration of high blood product ratios result in less impairment of coagulation factors and inhibitors and an therfore improved coagulation.

**Conclusion:**

The volume administered in severely injured patients decreased considerably during the last decade possibly supporting beneficial effects such as minimizing the risk of coagulopathy and avoiding potential harmful effects caused by blood product transfusions. Despite outstanding questions in trauma resuscitation, principle evidence merges quickly into clinical practice and algorithms.

## Background

Volume administration is one of the first therapeutic options in trauma care. While in 1993 Schwab and colleagues [1] already adapted the US Navy expression ‘damage control’ to trauma surgery, the administration of a significant amount of crystalloids was still common practice [2]. The benefits of delayed fluid resuscitation in hypotensive, bleeding patients were shown to improve patients’ outcome [3]. Subsequently, deleterious effects of excess crystalloid, such as abdominal compartment syndrome, acute respiratory distress syndrome or multiple organ failure (MOF), aroused attention [4, 5]. Non-indicated enhanced volume replacement therapy had furthermore been reported to be associated with increased mortality [6, 7].

At the same time the early posttraumatic coagulopathy has been recognized as an independent entity due to its high incidence at the time of emergency department (ED) admission [8–11]. In addition to endogenous triggers such as tissue trauma, inflammation and endothelial dysfunction, exogenous and iatrogenic factors like uncritical volume administration which contributes to acidosis and hypothermia (“vicious circle”) were described to enhance blood clotting disorder massively [12].

In order to reduce the amount of crystalloids, colloids were commonly administered in the treatment of haemorrhagic or septic shock. However, negative side effects such as acute kidney injury and impaired coagulation were observed [13]. Therefore, even after haemorrhage, the indication for colloids is questioned while the European medicines agency’s Pharmacovigilance Risk Assessment Committee (PRAC) inferred from recent studies that hydroxyethyl starch solutions (HES) must no longer be used to treat patients with sepsis or critically illness [14, 15]. However, hypertonic solutions are safe but do not show any benefits compared to crystalloids [16, 17].

The increasing awareness of the adverse effects of an incautious volume therapy has led to more restrained recommendations regarding volume replacement in pre-hospital care. Therefore, damage control principles were developed targeting a rapid control of surgical bleeding, an early use of red blood cells (RBC) and plasma, the limitation of excessive crystalloid use and permissive hypotension [18].

In the present study, the question is raised whether the academic awareness “doing more with less” has led to a reduced prehospital volume administration with balanced electrolyte solutions and colloids in the course of the last decade (2002–2012). Furthermore, it is investigated if an altered volume management has improved coagulation parameters of seriously injured patients and if consecutively less blood products with its potentially harmful effects such as single or multiple organ failure have been required.

## Methods

### The TraumaRegister DGU®

The TraumaRegister DGU® of the German Trauma Society (Deutsche Gesellschaft für Unfallchirurgie, DGU) was founded in 1993. The aim of this multi-centre database is an anonymous and standardized documentation of severely injured patients.

Data are collected prospectively in four consecutive time phases from the site of the accident until dis-charge from hospital: A) Pre-hospital phase, B) Emergency room and initial surgery, C) Intensive care unit and D) Discharge. The documentation includes detailed information on demographics, injury pattern, comorbidities, pre- and in-hospital management, course on intensive care unit, relevant laboratory findings including data on transfusion and outcome of each individual. The inclusion criterion is admission to hospital via emergency room with subsequent ICU/ICM care or reaching the hospital with vital signs and death before admission to ICU.

The infrastructure for documentation, data management, and data analysis is provided by the AUC - Academy for Trauma Surgery (AUC - Akademie der Unfallchirurgie GmbH), a company affiliated to the German Trauma Society. The scientific leadership is provided by the Committee on Emergency Medicine, Intensive Care and Trauma Management (Sektion NIS) of the German Trauma Society. The participating hospitals submit their data anonymously into a central database via a web-based application. Scientific data analysis is approved according to a peer review procedure established by Sektion NIS.

The participating hospitals are primarily located in Germany (90 %), but a rising number of foreign hospitals of other countries contribute data as well (at the moment from Austria, Belgium, China, Finland, Luxembourg, Slovenia, Switzerland, The Netherlands, and the United Arab Emirates). Currently, approx. 25,000 cases from more than 600 hospitals are yearly entered into the database.

Participation in TraumaRegister DGU® is voluntary. For hospitals associated with TraumaNetzwerk DGU®, however, the entry of at least a basic data set is obligatory for quality assurance.

The present study is in line with the publication guidelines of the TraumaRegister DGU® and registered as TR-DGU project ID 2013-057.

In this study, only patients from Germany were included in order to exclude variations in pre-hospital emergency and rescue systems among different countries. A full list of participating hospitals contributing to the TR-DGU can be found at www.traumaregister-dgu.de. All patients admitted were seen and treated by an emergency physician prior to hospital admission.

nclusion criteria for this analysis were as follows:

1. Age ≥ 18 years
2. ISS (Injury Severity Score) ≥ 16 points
3. Primary admission to the hospital (transfers excluded)
4. Standard documentation data record TR-DGU®
5. Hospitals participation for 5 or more years

Of the 100.489 patients entered into the data bank of the TR-DGU 2002–2012, a total of 23512 (23.4 %) matched with the inclusion criteria. The remaining numbers were excluded due to hospital transfers (*n* = 10989; 10.3 %), minor injuries (*n* = 42735; 42.5 %), injured children below 18 years (*n* = 1761; 1.72 %), data points that were edited into the short TR-DGU form (*n* = 15260; 15.2 %), TR-DGU participation for less than 5 years (*n* = 2132; 2.5 %), absence (*n* = 1328; 1.3 %) or unknown attendance (*n* = 1240; 1.2 %) of an emergency physician prior admission to the hospital and missing values for administered fluids and/or the red blood cells (RBCs) (*n* = 1532; 1.5 %). Similar to previous publications derived from the TR-DGU, shock is defined as systolic blood pressure less or equal (≤) 90 mmHg. Massive transfusion (MT) is specified as more or equal than 10 units of transfused RBCs until ICU admission. Ringer’s solution or 0.9 % NaCl are documented as crystalloid solutions; HES is documented as colloidal solution and HyperHES as hyperoncotic solution. Haemostatic drug therapy in the Emergency Department (ED) was documented since 2005; previous data are also not available for aPTT. Haemostatic drug therapy was defined as the following: administration of Factor VII, Prothrombin Complex Concentrate (PCC/PPSB), Fibrinogen and Tranexamic Acid.

### Statistics

Continuous and categorical values were calculated as overall values for the whole time period and separately for each year. If not presented graphically for each year, overall values are reported together with the first (2002) and the last (2012) value, or with the range (minimum - maximum) of yearly values. This retrospective analysis has a descriptive character. Formal statistical testing comparing all 11 years was avoided since even minor differences would result in highly significant results, due to the large sample size per year. However, in order to analyze potential trends and to estimate yearly changes, repeated linear regression analyses have been performed where the variable of interest was the dependent (i.e. predicted) variable, and the year of observation was the independent (i.e. the predictor) variable. The regression coefficient calculated for each year could then be interpreted as the approximate change per year. The *p*-value of this coefficient is presented as well. Interpretation of these results have to consider that this approach assumes a constant change over the time period considered which is not always the case.

We did not restrict our analysis to major bleeding cases since the application of volume has been used very deliberately in Germany, and prophylactic use of volume administration has been considered an easy and effective support of severely injured patients. Volume was given to at least 95 % of registry patients in the prehospital phase. The average annual values and their parallel increase or decrease over time are just observed associations, there is no proof of causality. A formal proof of the volume effect on lab values and transfusion rates would require a randomized trial. However, these measurements were made within a short period of time within each patient, on average within less than 1 h regarding initial lab values, and 2–3 h regarding transfusion. This would not allow to assume independence of the measurements. However, the degree of causation remains a matter of interpretation.

Data were analysed using SPSS statistical software package (Version 21, IBM Inc., Armonk, NY, U.S.A.)

## Results

**Fig. 1:**
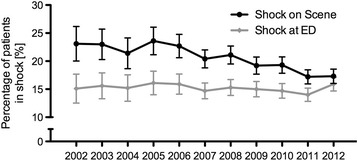
Percentage of patients in shock (≤90mmHG systolic blood pressure) during the prehospital phase and by the time of arrival in ED

A total of 23512 patients from the TR-DGU® met the inclusion criteria for further analysis. General demographics and injury description as well as their respective changes per year are shown in Table 1. While most of the trauma mechanisms did not differ during the observation period, motor vehicle accidents (MVA) decreased from 40.4 % in 2002 to 29.7 % in 2012 whereas low falls (<3 m) increased from 6.7 to 11.9 %. The proportion of severe head injuries (AIS head ≥3) remained constant while severe abdominal injuries (AIS abdomen ≥3) occurred significantly less often (Table 1). The mean ISS was 29 points and did not change over 11 years, while the NISS increased slightly (Table 1). On scene, fewer patients were in shock (23.1 % in 2002 vs. 17.3 % in 2012) while the number of patients in shock admitted to ED remained constant (Fig. 1).

**Table 1:** General demographics, characteristics of injury and key features of volume management and their respective change per year

		All patients	Value 2002	Value 2012	Change per year	*p*-value
Demographics	Age (years; mean (min-max))	47.0 (43.8–50.3)	43.8	50.3	0.83	<0.001
	Male (%; mean (min-max))	72.9 (71.9–74.3)	73.4	71.9	−0.2	0.11
Injury assessment	Penetrating trauma (%; mean (min-max))	4.2 (3.5–5.6)	4.2	4.2	−0.1	0.08
	AIS Head ≥3 points (%; mean (min-max))	56.0 (53.0–59.9)	55.4	53.0	−0.3	0.01
	AIS Thorax ≥3 points (%; mean (min-max))	58.9 (54.0–61.1)	59.8	59.9	−0.1	0.15
	AIS Abdomen ≥3 points (%; mean (min-max))	19.6 (17.3–25.4)	25.4	17.3	−0.9	<0.001
	ISS (points; mean (min-max))	28.9 (28.3–29.6)	28.6	28.3	−0.08	0.003
	NISS (points; mean (min-max))	35.1 (33.4–36.2)	33.6	34.6	0.04	0.001
Volume administration	Total prehospital volume (ml; mean (min-max))	1282 (1039–1790)	1790	1039	−73.7	<0.001
	Prehospital crystalloid fluids (ml; mean (min-max))	931 (1156–837)	1156	849	−30.6	<0.001
	Prehospital colloid fluids (ml; mean (min-max))	317 (169–574)	574	169	−39.3	<0.001
	Prehospital hyperoncotic fluids (ml; mean (min-max))	34.2 (20.0–59.6)	59.6	20.0	−3.5	<0.001
	Total volume at ED (ml; mean (min-max))	2030 (1345–3191)	3191	1416	−222	<0.001
Blood products	RBC units/patient, all (n; mean (min-max))	2.1 (1.6–3.8)	3.8	1.6	−0.21	<0.001
	RBC units/patient, if transfused (n; mean (min-max))	8.1 (9.8–7.4)	9.8	7.6	−0.22	<0.001
	Patients with ≥1 RBC (%; mean (min-max))	26.0 (18.1–38.7)	38.7	20.6	−2.0	<0.001
	Patients receiving MT (%; mean (min-max))	7.1 (4.8–13.4)	13.4	5.2	−0.8	<0.001

**Fig. 2:**
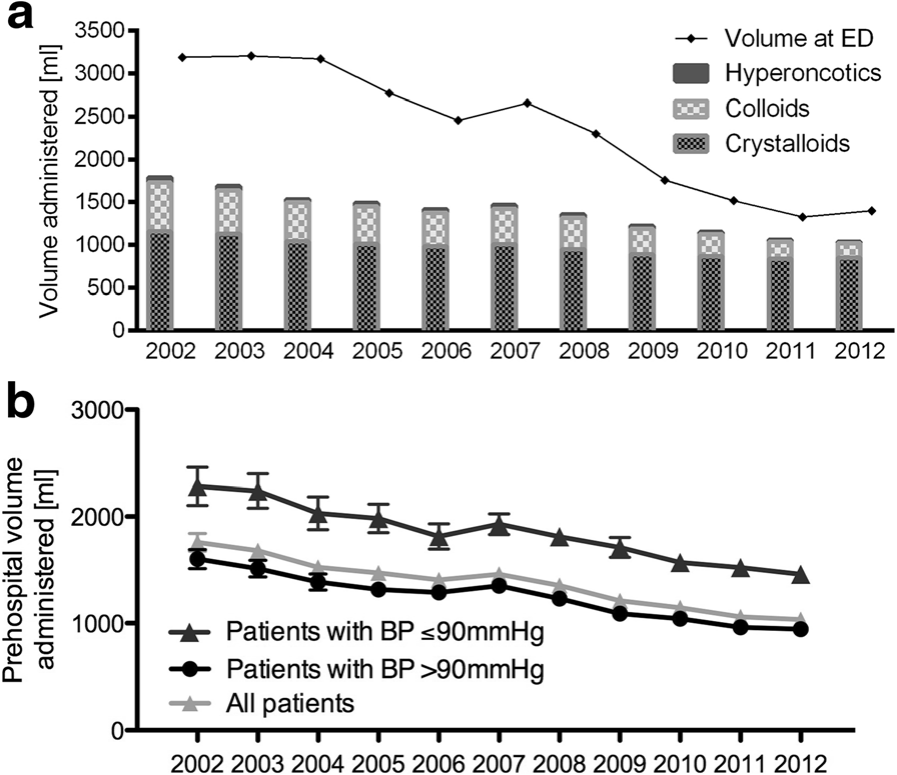
**a** Mean prehospital fluid administration in millilitres (ml) per year for crystalloids, colloids and hypertonic solution and average amount of volume given at the emergency department. **b** Administered prehospital volume for patients depending on first measured blood pressure (shock defined as systolic blood pressure ≤ 90 mmHg)

The analysis of the prehospital volume administration between 2002 and 2012 showed that 22575 patients (96.0 %; min - mix 95.0–96.9 %) received prehospital intravenous (i.v.) fluids by the emergency physician. If volume was given, most patients (*n* = 22110; 97.9 %) received crystalloids. The use of colloids and hyperoncotic solutions, which were usually given in combination with crystalloids, decreased drastically over time (colloids: 69.6 % in 2002 to 28.5 % in 2012; hyperoncotic fluids: 16.4 % in 2002 to 6.0 % in 2012). In contrast, the number of patients receiving crystalloids stayed constantly high (range 96.6 to 98.8 %) in the period presented. The mean volume administered per year subdivided into fluid types, respectively, is shown in Fig. 2a and Table 1.

The average amount of total volume administered decreased from 1790 ml to 1039 ml (Fig. 2a). Comparing the amount of volume administered to patients with and without shock, a steady decrease can be observed in both groups (Fig. 2b). As expected, more volume was administered to patients in shock. Similar to the trend of reduced prehospital volume administration, the volume administered in the ED dropped from 3191 ml in 2002 to 1416 ml in 2012.

**Fig. 3:**
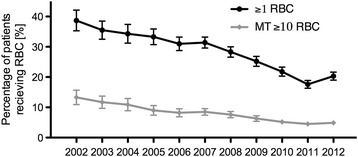
Percentage of patients receiving ≥1 RBC and massive transfusions (≥10 RBC’s within 24 h) per year

**Fig. 4:**
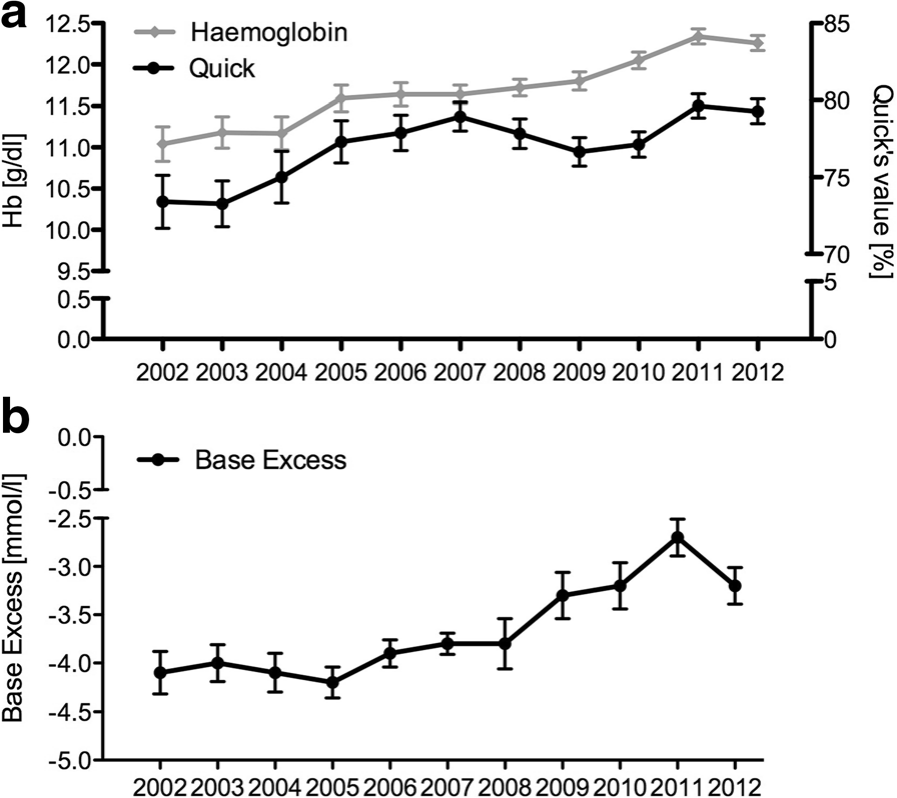
Laboratory results in first blood analysis. **a** Hb and Quick’s value by the time of arrival in the emergency department. **b** Base Excess (BE) by the time of arrival in the emergency department

Over the time observed, the rate of trauma patients who received prehospital catecholamines inclined from 9.2 to 13.0 %. The proportion of patients receiving catecholamines at the ED stayed constantly around 30 %. At the same time, fewer patients received RBCs (38.7 % in 2002 vs. 20.6 % in 2012; Fig. 3). If transfused, the number of RBC units administered decreased per patient by -0.22 units per year (9.8 RBCs in 2002 vs. 7.6 in 2012). In contrast units of fresh frozen plasma (FFPs) were given to an equal extent, mirroring nearly a 1:1 RBC:FFP ratio in 2012 (6,7 FFP in 2002 vs. 6,1 FFP in 2012). The lowered administration of blood products was further accompanied with less MT that even more than halved (13.4 % in 2002 vs. 5.2 % in 2012). Haemoglobin and Quick’s values increased at admission and the abnormality of the mean Base Excess (BE) declined (Fig. 4). Since the start of recording in 2005, there is a steady rise in the use of haemostatic medication from 10.3 to 18.3 %.

## Discussion

More than two decades passed by since physicians and researchers started to realize the deleterious effects of excessive administration of crystalloids [4, 5].

Although it is well known and taught among emergency personnel that factors such as age >80 years, ISS ≥16, GCS <8, hypotension and excessive administration of i.v. fluids promote patients mortality, [19] it has not been proven if evidence obtains clinical practice.

The period analysed in the present study includes the point in time when rising interest and understanding among experts in the role of hypothermia, coagulopathy and acidosis aroused [4, 5]. Since uncritical volume administration was identified to enhance this vicious circle, a steady decrease of the administered volume after severe trauma can be observed in both, the prehospital use and in ED.

Collecting data in a databank such as the TR-DGU® enables analysis of trauma cases and therapeutic strategies accordingly. As expected with an aging society, patients enclosed in this study grew 0.83 years older per year on average for the timeframe observed, which might at the same time explain the increased rate of low falls below three meters. Improved road safety results in a lower percentage of major road accidents. Nevertheless, also the number of high falls of more than three meters height increased in the period observed. Despite these changes in injury mechanisms, the mean ISS and NISS stayed consistently high and could therefore not cause these differences in volume management. A higher number and a higher severity of injuries of the extremities and pelvis might equalize the decrease of AIS abdomen.

During the last years the evidence even more mounted that aggressive crystalloid administration rather causes a worsening acidosis, may trigger an early inflammatory response, and also entails an substantial increase in morbidity such as Acute Respiratory Distress Syndrome (ARDS), MOF, compartment syndromes and surgical site infections [20, 21]. Although there is less doubt about i.v. fluid administration in trauma patients to maintain tissue perfusion, there is no evidence about any volume threshold for trauma patients [22]. Among others, [23] the PROMMTT Study Group showed that pre-hospital fluid administration is not associated with increased systolic blood pressure but decreases in-hospital mortality [24]. In contrast, Geeraedts et al. described, that in a cohort of hypotensive trauma patients, prehospital volume therapy was associated with a decrease of the likelihood of shock defined as shock index ≥ 1 [25]. Interestingly, patients in this study received decisively less volume (median, min to max: 0.5 L, 0–8.1 L) compared to our data. The difference might be caused by the fact that Geeraedts et al. included all hypotensive trauma patients, regardless of the injury severity. However, this study underlies the importance of cautious pre-hospital volume replacement.

Furthermore, HAES should not be used in most cases except therapy refractory haemorrhagic shock and possibly penetrating trauma due to less renal affection and decreased lactate levels [26]. Unfortunately, information about the type and concentration of Hydroxyethyl starch are not available, in order to accomplish a more detailed analysis. However, according to the recommendations, we can clearly file the reduced over all use in our data. In comparison of the three different fluids administered the most notable reduction can be seen in colloids.

Although fewer patients were in shock on scene and the prehospital treatment differed decisively, the rate of patients in shock remained steady by the time of arrival at the ED. However, improved BE at the time of admission at ED might indicate a better tissue perfusion despite unaltered low BP. Apparently the decrease in volume administered was potentially compensated by catecholamines given to a greater extend. In trauma patients, the administration of catecholamines recruits unstressed blood volume and might maintain blood pressure [27]. But despite numerous arguments in favour of early vasopressor use, there is still a lack of evidence derived from prospective studies [28]. Some authors even associate vasopressor exposure with an increased mortality [29, 30]. Since catecholamines might influence the presented data masking a true intravascular volume deficit, [31] we observed indeed a reduced extend of hypovolemic shock, mirrored by the reduced BE by the time of emergency admission [32]. However, the continuous reduction of BE interferes with the unaltered rate of patients with systolic blood pressure below 90 mmHg. Although Eastridge et al. showed that mortality starts to increase with a systolic BP below 110mmHG in patients without brain injury, we defined shock as a BP below or equal 90 mmHg in order to include patients in severe shock [33]. The question remains, if the definition of shock using solely the systolic blood pressure is applicable in this study, although BE values are usually not compiled preclinical.

As described in the current literature, inadequate volume administration and endogenous factors such as tissue hypoperfusion, inflammation and the acute activation of the neurohumoral system contribute to coagulopathy [21]. Although it has been described that crystalloids given even increase thrombin generation [34] and that low quick values are not necessarily attend with diminished thrombin generation [35], minimizing the iatrogenic contribution to the development of a TIC might have resulted in a higher Quick’s value and shortened aPTT.

Furthermore, the use of haemostatic drugs increased within the observation period. The administration of Tranexamic Acid (TXA) within 3 h after injury significantly reduced the all-cause mortality and in particular due to bleeding [36]. Subsequently, an early prehospital use of TXA is already recommended in the updated European guidelines [17]. Nevertheless, benefits of prehospital administration of TXA are widely lacking but a prospective, blinded study is currently underway. The study design was recently published scrutinizing this issue [37].

A further target to avoid coagulopathy is discussed by substitution of fibrinogen as fibrinogen depletes early after trauma. The effect of prehospital fibrinogen concentrate administration is the subject of an on-going prospective investigation [38]. Since the results of these studies might support the prehospital use of both, fibrinogen and TXA, the administration of haemostatic drugs is likely to incline within the next years.

The changes observed in volume therapy might have reduced haemodilution potentially resulting in an increase of the Hb value, which might explain that in 2012 the number of patients receiving blood products halved in comparison to 2002. Correspondingly the percentage of patients receiving MT halved. Already Geeraedts et al. showed, that the likelihood of receiving blood transfusion increases dramatically associated with increasing prehospital volume [25]. Thereby, the development during the last years is certainly along the right lines to avoid mass transfusions as blood products may imply negative secondary effects, such as single or multiple organ failure and sepsis [39, 40]. Besides volume administration, the decreasing rate of MT has multiple reasons. Transfusion strategies have become more restrictive and ratio based to avoid over transfusion and diminish negative effects caused by blood products [41, 42]. Furthermore the updated European guidelines recommend a target-haemoglobin of not more than 7 to 9 g/dl (1c) [17]. In addition, high blood product ratios result in dilution of coagulation factors and inhibitors, prolonged PTT and increased INR and reduced functional coagulation in viscoelastic testing [43]. The quantity of RBCs and FFPs transfused nearly approached a 1:1 ratio, which reflects principles of damage control resuscitation [44] and the recommendations of the current European guidelines [17]. However, the amount of FFPs transfused only declined slightly.

Certainly as this study is a retrospective register analysis, there are limitations such as susceptibility to observer bias, bias because of selective survival information, incomplete or inaccurate information and dependency on respondents. Therefore, only associations and no causalities can be derived from the underlying data. Still, the TR-DGU® enables illustration of consistency or changes in therapy or therapeutically actions.

## Conclusion

The volume administered in severely injured patients decreased considerably during the period observed. In addition to an increased use of haemostatic drugs and goal directed transfusion protocols the need for transfusion is likely to decline further. Although having identified principal drivers of coagulopathy and implementing evidence based algorithms, it still remains unclear, which basic amount of pre-hospital volume therapy is needed. Therefore, subsequent research is inevitable. Despite outstanding questions, principle evidence in volume management has rapidly merged into clinical practice and algorithms during the last years.

## Abbreviations

DGU: Deutsche Gesellschaft für Unfallchirurgie (German Trauma Society)
MT: massive transfusion
MTP: massive transfusion protocol
MVA: motor vehicle accident
PRAC: European medicines agency’s Pharmacovigilance Risk Assessment Committee
TXA: Tranexamic Acid
i.v.: Intravenous
pRBCs: packed red blood cells
FFP: fresh frozen plasma
PLT: Platelet concentrates
Hb: Haemoglobin value
aPTT: activated partial thromboplastin time
PPSB/PCC: Prothrombin Complex Concentrate
ED: Emergency Department
TIC: trauma induced coagulopathy
BP: blood pressure
